# Climate Change and Major Pests of Mediterranean Olive Orchards: Are We Ready to Face the Global Heating?

**DOI:** 10.3390/insects12090802

**Published:** 2021-09-08

**Authors:** Alice Caselli, Ruggero Petacchi

**Affiliations:** BioLabs, Institute of Life Science, Scuola Superiore Sant’Anna, Piazza Martiri Della Libertà 33, 56127 Pisa, Italy; ruggero.petacchi@santannapisa.it

**Keywords:** *Bactrocera oleae*, climate emergency, Mediterranean Basin, *Olea europaea*, olive pest

## Abstract

**Simple Summary:**

The phenomenon of climate change affects the entire world, especially the most vulnerable areas such as the Mediterranean. Since the olive growing is one of the main economic sources for the Mediterranean countries, investigations on olive pests under global heating are necessary. Nowadays, knowledge on the topic is scarce, and nothing is known about the effects of climate change on olive pest parasitoids and predators. This information could be fundamental to understand the phenomena of pest outbreaks that are spreading in the Mediterranean olive orchards. The use of prevention tools (e.g., monitoring, prediction models) may help in controlling olive pests under a climate change scenario.

**Abstract:**

Evidence of the impact of climate change on natural and agroecosystems is nowadays established worldwide, especially in the Mediterranean Basin, an area known to be very susceptible to heatwaves and drought. *Olea europaea* is one of the main income sources for the Mediterranean agroeconomy, and it is considered a sensitive indicator of the climate change degree because of the tight relationship between its biology and temperature trend. Despite the economic importance of the olive, few studies are nowadays available concerning the consequences that global heating may have on its major pests. Among the climatic parameters, temperature is the key one influencing the relation between the olive tree and its most threatening parasites, including *Bactrocera oleae* and *Prays oleae.* Therefore, several prediction models are based on this climatic parameter (e.g., cumulative degree day models). Even if the use of models could be a promising tool to improve pest control strategies and to safeguard the Mediterranean olive patrimony, they are not currently available for most *O. europaea* pests, and they have to be used considering their limits. This work stresses the lack of knowledge about the biology and the ethology of olive pests under a climate change scenario, inviting the scientific community to focus on the topic.

## 1. Introduction

Climate emergency is the major environmental global challenge of this century, and anthropogenic activities are the most significant driver of this phenomenon [1,2,3]. Dramatic data reveal that the carbon dioxide concentration has increased from 280 ppm in the preindustrial period to 401 ppm in 2015. Furthermore, an increase in temperature of about 0.85 °C has been recorded since the 19th century, and future projections hypothesize the average temperature will increase further [2,4]. Indeed, the global human population growth entails an ever-rising demand for food, placing pressure on the regulation of agroecosystem services [4,5,6]. The agriculture sector is one of the major contributors to climate emergency, and paradoxically, it is also the most afflicted by its worst impacts [6]. As a result of this imbalance, pest populations may become alarming, exceeding the economic thresholds of insect pests [2]. Climatic change directly interferes in insect survival, fecundity, voltinism and dispersal, and among climatic parameters, temperature is the most influential since insects are heterothermic [2,7,8]. Abiotic factors, such as temperature and humidity, can alter the coadaptation relationship between a host and its pest, resulting in pest outbreaks [9,10]. The capacity of a pest to face climatic variation is strictly related to the physiology of its host plant(s) [11,12] and to the adaptation of its natural enemies and their fitness, as temperature can affect the host searching behavior and the parasitization efficacy [13]. Insects may respond to climate change in several ways, with modification in phenology and distribution, influencing the composition of the community and causing great disorders on population dynamics (e.g., the size of swarming populations, the alteration of voltinism) [2,7,14,15].

The Mediterranean Basin is an area highly vulnerable to climate change, and observed rates for most climatic parameters exceed global trends [16,17]. Additionally, southern Mediterranean countries are expected to be the most afflicted by heatwaves and drought, and a huge backlash on agriculture productivity is predictable [18,19]. *Olea europaea* L. is one of the oldest permanent crops of the Mediterranean, where it has an essential socioeconomic and ecological relevance [19,20,21,22,23,24]. Nearly all (95%) of global olive oil demand is satisfied by the southern European countries; among them, Spain is the most devoted to oil production, followed by Italy, Greece and Portugal [23,25]. This high productivity is explained by the perfect adaptation of the olive tree to the Mediterranean climate due to its tolerance to drought conditions [18,23,26,27]. However, future projections of climate change forecast challenging perspectives for olive growers since temperature is increasing and water availability is becoming a serious problem for Mediterranean agriculture, particularly on European islands (e.g., Corsica, Sardinia and Sicily) that are expected to be highly susceptible to desertification [19,28]. *Olea europaea* is considered a sensitive indicator of the climate change degree since its phenology is strongly related to the temperature trend [25,29,30]. High temperatures may cause a default of *O. europaea* chilling requirement (450 h of chilling below 7.3 °C) and consequent problems in flowering and fruit bud development [31,32]. In addition to temperature, other abiotic factors directly influence olive productivity, such as precipitation, solar radiation, relative humidity and wind [25]. Furthermore, climatic parameters interfere in the manifestation of olive pests and diseases [25]. The set of organisms potentially detrimental to *O. europaea* is composed of more than 255 species, including insect pests, mites, nematodes and pathogenic microorganisms [33,34]. In the Mediterranean Basin, the yield loss caused by insects alone is estimated at about 15% of production per year, and the majority of this drop is caused by the key olive pest *Bactrocera oleae* (Rossi) (Diptera: Tephritidae) [34]. Furthermore, other major secondary pests (e.g., *Prays oleae*) contribute locally or occasionally to the yield decline [34]. Insects can also play the role of pathogen vectors affecting *O. europaea* indirectly. For instance, in the Salento peninsula (Italy), the main cause of the olive quick decline syndrome is the bacterium *Xylella fastidiosa* transmitted between olive plants by the spittlebug *Philaenus spumarius* L. [35]. In this scenario, the ability to predict pests’ cycle and damage risk is a promising tool for programming a proper control strategy. To have a prediction as close as possible to the reality, the use of physiologically based models that include the effects of weather, the assessment of previous pest population densities, the biology of the pest and the interactions between key species is required [28,36,37,38,39,40].

Although olive growing is one of the main sources of income for Mediterranean agriculture [41], few studies are nowadays available concerning the consequences that climate change may have on major olive pests. This review examines the current knowledge on the topic, focusing on the possible adoptable strategies for dealing with future challenges and perspectives in a climate change scenario.

## 2. Climatic Consequences on the Key Olive Pest *Bactrocera oleae*

Worldwide, the exclusive key insect pest of the olive tree is the tephritid fly *B. oleae* [22,34,42,43,44]. *Bactrocera oleae* is a monophagous pest on the genus *Olea* that causes direct damage to olive yield since its larval stages feed on drupe pulp [43,45,46]. It causes serious economic losses that have been estimated at more than USD 1 billion per year in the Mediterranean alone [47]. In this area, *B. oleae* can complete several generations that vary from one to four depending on temperature and area characteristics (e.g., elevation, distance to sea) [40,48,49]. Among the environmental factors, temperature is the key parameter influencing *B. oleae* phenology and the relation with *O. europaea* [50]. High temperatures in summer induce *B. oleae* mortality and slowdown on pest activity, since adult physiological processes cease at 35 °C [51,52]. However, other patterns such as weather conditions, season extension and crop–pest synchrony impact temporal changes in pest abundance [40,53]. The tight relation that links *B. oleae* to *Olea* species [42] makes this system a proper scenario for studying the climatic change in the Mediterranean Basin [28].

### 2.1. Prediction Models of B. oleae Population Dynamics

The global climate change may influence insects’ population dynamics and pest outbreaks [40]. The ability of *B. oleae* to fly for long distances and the poor knowledge on the overwintering generation may influence the monitoring of the pest and consequently the prediction of infestation risk [49]. In this scenario, the use of prediction models is essential to improve pest control strategies, deal with environmental impact and ameliorate product quality [49,54,55]. Models based on long-term datasets, concerning both insect population dynamics and weather parameters, are relevant for organizing proper pest control strategies [40]. Even if *B. oleae* is the major insect pest of the olive tree worldwide, few studies have been currently done about predictive modeling of olive fly population dynamics, particularly in a climate change scenario. Below, models based on cumulative degree day, *B. oleae* physiology, endogenous and exogenous factors influencing *B. oleae* demography and machine learning models are analyzed. 

#### 2.1.1. Cumulative Degree Day Models

*Bactrocera oleae* evolves from pupae to adult when the cumulated degree day (CDD) reaches the value of 379.02 from oviposition, which usually takes place in October (base temperature of 8.99 °C) [40,49,56]. The heat unit accumulation by CDD has always been used as a temperature-dependent method to predict the adult emergence [49], even if an error of 10–15% is estimated [49,57]. In order to reduce errors in the CDD model, accurate calibration of the starting date is needed, and proper monitoring of *B. oleae* flight during the previous winter and spring seasons is essential [49]. Furthermore, the availability of long-term insect monitoring greatly influences the prediction quality [58]. In Liguria (northwest Italy), Petacchi et al. (2015) [49] demonstrated that the CDD model, supported by GIS approach and agrometeorological regional network, gave a reliable prediction of *B. oleae* emergence, highlighting the *B. oleae* diversity at the local scale and the strong coastal influence in pest distribution. According to Volpi et al. (2020) [59], the olive fruit fly finds advantageous conditions in coastal areas characterized by cool summers and mild winters, and the probability of infestation becomes low if the olive is cultivated far from the coast. The output of the CDD model was mapped with regression correlation, providing a precise description of *B. oleae* diversity and reporting the high spatial climatic variability of Liguria [49]. Additionally, Marchi et al. (2016) [40] described an important relationship between the degree of *B. oleae* infestation and temperature-based indices in Tuscany (central Italy), using 13 years (2001–2014) of monitoring data and a CDD model. In this temporal span, the highest attack by *B. oleae* was recorded in 2007 and 2014, years in which mild winters were observed. Indeed, in the last years, an increase in winter temperatures with few frost days has been registered in the Tuscany region [40,60]. These conditions usually cause a reduction in pest mortality and an acceleration in recovering from overwintering [59,61]. Furthermore, during summer 2014, temperatures rarely overpassed the thermic threshold of 35 °C. In this context, adult physiological activities remained undamaged, and great consequences on olive yield were reported [40]. The CDD model has also been used in northeast Portugal to predict the second generation of *B. oleae*, the most threatening in the Trás-os-Montes region, using data from 2005 to 2008 [62]. The model was suitable for predicting in advance *B. oleae* infestation both in 2006 and 2008. In 2007, the same model was not so efficient, probably in consequence of abnormal low summer temperatures (average 20.7 °C) [62]. Despite this unusual condition, the model was demonstrated to be a potential tool for *B. oleae* management, allowing the prior identification of the second-generation activity and consequently the estimation of the infestation risk [62].

#### 2.1.2. Machine Learning Models

Machine learning (ML) algorithms are used for various purposes (e.g., data mining, image processing, predictive analytics) and allow the users to manage complex datasets and target trend analysis [59,63]. In agronomical studies, they are generally employed to build predictive models from regression or classification analysis depending on the variables [64]. For this purpose, large datasets are required over several years [65]. Volpi et al. (2020) [59] described an ML model based on a long-running sampling network (2002–2019) to predict, in Tuscany, the occurrence of the first summer generation of *B. oleae*. The algorithm selected for the model was greatly able to distinguish both the presence and the absence of the infestation, reaching an accuracy of 85% and 78%, respectively [59]. The model properly identified the mechanism that drove the occurrence of the olive fly summer generation, highlighting a good ability as a preventive tool in IPM of *B. oleae*. However, it did not show a clear relationship between summer temperatures and the olive fruit fly infestation [59]. 

Among ML techniques, the maximum entropy (ME) is a statistical–probabilistic technique that, except for its complex math, does not require high precision and huge datasets to accurately estimate species distributions [66]. It has been used to model the climatic suitability of *B. oleae* in the Iberian Peninsula and its probability of occurrence, determining a priori the presence or the absence of *O. europaea* (see Benhadi-Marín et al., 2020 [67]). In the model, the climatic suitability of the olive fruit fly was negatively influenced by high precipitation values recorded in the coldest quarter, and precipitation of the driest month agrees with the adaptation of the olive fly and the olive tree to the Mediterranean drought occurring in summer since it is in harmony with *B. oleae* bioecology [67]. Limiting environmental factors for both *B. oleae* and the olive tree are the main drivers influencing the habitat requirements for olive fly survival [67]. Additionally, temperature deeply contributes to the model, predicting a strong decrease in *B. oleae* suitability at a mean diurnal range >10 °C and an optimal for the mean temperature of the coldest quarter at 4.5 °C [67].

#### 2.1.3. Physiologically Based Demographic Models

The management of a pest must include realistic estimates about its phenology and potential distribution in time and space and an evaluation of the damage [68]. These estimates are based on the multifactor time-varying complexity of pest systems, which are often tricky to assess, particularly in a climate change scenario [69]. For these purposes, mechanistic models, such as physiologically based demographic models (PBSMs), could be successfully used [70]. Gutierrez et al. (2009) [51] described the Italian distribution and abundance of olive and *B. oleae* using a PBDM under observed weather, considering 84 locations covering the whole peninsula, and climatic data from 1999 to 2005. The model created three warming scenarios by increasing daily mean temperature (+1 °C, +2 °C, +3 °C) and assuming all other variables as unaltered. The model estimated an increase in olive yields in the entire Italian territory, especially in the northern currently inhospitable areas (e.g., Po Valley), in all warming scenarios. However, some reductions in olive tree adaptability are predicted in southern areas due to excessively hot temperatures [51]. The PBDM estimated higher damages due to *B. oleae* with a 1 °C increase in temperature, particularly in northern Italy since the weather was predicted favorable for the species and also for the olive tree. Increases of +2 °C and +3 °C, instead, seem to cause inhospitable conditions for olive fly reproduction and survival, especially in the southern areas of the peninsula [51].

#### 2.1.4. Model Based on Exogenous and Endogenous Factors Influencing Insect Population Dynamics

Insect population dynamics can be well investigated when global climatic indicators are considered together with local weather conditions [71]. Among global indicators, the North Atlantic Oscillation (NAO) is an important factor influencing plant and animal populations in the Mediterranean Basin [72]. Ordano et al. (2015) [73] investigated and modeled the joint role of exogenous (e.g., local climatic factors, NAO) and endogenous (e.g., intrinsic population dynamics) factors involved in the autoregressive process and population dynamics of *B. oleae* in five locations within Palestine and Israel. The model revealed that the main exogenous driver in all populations was the local climatic variation measured as night land surface temperature, while NAO was influential in only one of the studied populations [73]. The same population was also significantly influenced by olive fruit availability [73]. However, despite the strong influence of exogenous factors, the model indicated endogenous factors as the main driver influencing *B. oleae* population dynamics since it showed recurrent olive fly infestations revealing density-dependent population feedback [73].

#### 2.1.5. Considerations on the Reliability of Predictive Models

Predictive models are based on associations between climate and natural distribution of a species, and they are focused on the conditions that keep the population alive and viable [74,75]. They are widely used in applied ecology to predict the future distribution of a species in a climate change framework [76]. Ecological niche models, such as the already cited ME model, are used in olive orchard context to provide future scenarios not only on insect pests but also on the epidemiology of pathogens, such as *X. fastidiosa* [77], and on *Olea* spp. future distribution [78]. However, predictive models have been recently questioned since they do not take into account many factors (e.g., biotic interactions, evolutionary change, dispersal ability) that together with the climate are involved in determining the distribution of a species [74,79]. Furthermore, species distribution models that are based entirely on contemporary realized distributions are considered potentially misleading for previsions in a climate change context [80]. Moreover, data must be collected according to a structured sampling design in order to avoid limits in drawing deductions on species distribution [76]. Despite the limits reported above, ME is one of the most popular predictive models since its simulation precision is greater than that of other niche models. Moreover, it has a short operation time, and it can also be used with small samples [79]. In general, the bioclimate predictive models can provide useful approximation about the impact of climate change on biodiversity and species distribution. Indeed, scientists must consider that the spatial scale at which these models are used is essential to interpret the results. Furthermore, to avoid misleading interpretation, the model’s output should be read taking into account the limitations involved in the selected model [74].

### 2.2. Climate Influence on B. oleae Parasitoids and Predators

The current knowledge about the influence of climatic factors on *B. oleae* parasitoid and predator complex is extremely patchy, despite the high interest in olive fly natural enemies as bio-controllers [22]. Among *B. oleae* parasitoids, *Psyttalia concolor* (Szépligeti) (Hymenoptera: Braconidae) has been the most studied [81,82]. It was introduced in Europe in the early 1900s as a natural enemy of the olive fruit fly, but unsuccessfully [82,83]. Low winter temperatures may contribute to this failure, since *P. concolor* survival is negatively influenced by cold [82]. Furthermore, the climate breakdown may limit the success of *B. oleae* parasitoids [44]. Recently, Abd El-Salam et al. (2019) [44] described the influence of temperature and relative humidity on *P. concolor* survival in two Egyptian localities vulnerable to climate change. The study showed that the adverse change of temperature and relative humidity negatively influenced the relationship between the young stages of *B. oleae* and their parasitoid *P. concolor* [44]. Furthermore, temperature had a key role in determining *P. concolor* survival compared to relative humidity [44].

Soil arthropods (e.g., carabids, staphylinids, ants, spiders, opilionids, centipedes, earwigs, chilopods) are included in the predator complex of *B. oleae*, since the olive fly larvae leave the drupe to pupate in the soil before the winter [22,84,85]. The soil environment is highly dynamic, both because it hosts a huge number of plants and arthropods and because it is susceptible to changes in moisture, temperature and fluctuating redox states [86]. Even if most climate studies refer to the atmospheric conditions, climate change strongly influences soil characteristics (e.g., temperature, soil organic carbon) [87,88] and, consequently, arthropods dwelling in the ecosystem. However, to the best of our knowledge, no studies have been currently carried on about climate heating and soil predators of *B. oleae*.

### 2.3. Control Strategies of B. oleae under Global Warming

Climate change is one of the main factors that contribute to the use of pesticides and influences their behavior in the environment (e.g., transformation, degradation, volatilization, runoff, leaching) [89]. Historically, the control of *B. oleae* has been mainly based on the use of chemicals, even if it has changed over time [33,90]. Nowadays, since global heating influences the biology and distribution of *B. oleae* [50], its control methods must be adjusted according to this phenomenon. In autumn, the current extension of the period in which temperatures are favorable to *B. oleae* oviposition (>12 °C) increases the risk of yield loss for Mediterranean olive producers [91]. Marchini et al. (2017) [92] declared that olive fly females can complete one generation in spring, adding new evidence on the reproductive behavior of this species and pointing out the necessity of proper control strategies. Accordingly, preventive adulticide treatments (e.g., attract and kill techniques with canopy traps, bait traps) that cause a decrease in *B. oleae* reproductive activity are recommended to reduce *B. oleae* population in the following summer when olive fruits are set [92].

**Figure 1:**
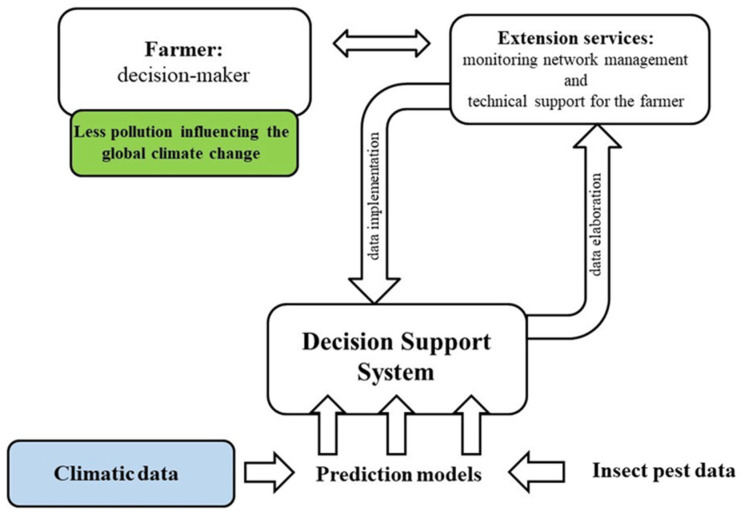
Long-term climatic data are the basis of insect pest prediction models together with insect data obtained by proper monitoring [40]. The decision support systems (DSSs), which bridge the gap between prediction models and extension services, have the task of technically supporting farmers in olive orchard management. In order to abandon scheduled treatments and then to avoid the risk of carrying out unnecessary chemical uses, farmers need a good scenario of the current status of the pest and its potential future trend. As a consequence of a conscious olive orchard management, positive outcomes on the environmental impact and global heating can be recorded (e.g., less air pollution) [49,54,55,96].

Moreover, the recent discovery of insecticide residues in olive oil and in the environment and the growing resistance to chemicals encourage the shift to eco-friendly control methods (e.g., botanical insecticides, insect growth regulators, semiochemicals) and conservation biological control programs (e.g., the enhancement of generalist predators) [33,93]. The implementation of a decision support system (DSS), together with reliable monitoring of the pest, allows bridging the gap between prediction models and extension services and technically supports the olive farmers in suggesting suitable management for the orchard [40]. In accordance with this, scheduled calendar treatments are abandoned, and chemical applications are reduced in time and space in favor of integrated pest management [40,94] (Figure 1). Furthermore, the recent development of advanced data process technologies allows monitoring and managing insect pests following an ecofriendly approach [90]. For instance, precision agriculture in olive fly control drives the chemical application directly on the hot spot area, limiting the treatment just to a few trees per orchard [90,95]. This causes a decrease in pesticide application and consequently a limitation of environmental pollution, since the chemical drift is reduced and the use of fossil fuel (CO_2_ emission) for the sprayer machines is limited [95,96]. Therefore, in the olive orchard, the adoption of new technologies together with alternative and eco-friendly control strategies might contribute to limit global heating thanks to a more sustainable use of pesticides and a reduction in air pollutants, limiting the carbon dioxide emission overall.

## 3. Influence of Climate on Some of the Major Secondary Pests of *O. europaea*

### 3.1. Olive Moths

The ecology and phenology of olive lepidopterans are strongly conditioned by weather conditions, characteristics of the host and the presence of antagonists [97]. Temperature is the most influential climatic parameter on the correlation between *O. europaea* and its moth pests [97]. Among the numerous moths linked to *O. europaea*, *Prays oleae* (Bernard) (Lepidoptera: Praydidae) is a major pest in all Mediterranean olive-growing scenarios [97]. The intensity of its attack is related to local climate conditions and olive phenology. In Morocco, coastal areas are more vulnerable to *P. oleae*, since temperatures are mild and humidity is important, while warmer and less humid regions do not advance *P. oleae* survival [97]. Morocco is one of the most vulnerable regions of the North Africa states to climate change because of its sensitivity and its limited adaptive capacities [98]. In this fragile framework, in which the gravity of *P. oleae* attack fluctuates each year depending on the climate [99], the use of prediction models could strongly contribute to *P. oleae* control. 

In Greece, the polyphagous moth *Euzophera bigella* (Zeller) (Lepidoptera: Pyralidae) was noticed for the first time infesting the olive tree in 2011. Strong infestations were recorded in the province of Amphipolis with few cases of tree death [100]. The reasons for this outbreak were not investigated; however, Simoglou et al. (2012) [100] hypothesized a combination of climate alterations as the main driver.

*Palpita vitrealis* (=*P. unionalis*) (Rossi) (Lepidoptera: Pyralidae), also known as the jasmine moth, is an important pest of young olive plantations in several Mediterranean regions (e.g., Italy, Algeria) [101,102]. It feeds on new buds and leaflets causing shoot malformation and alteration of the vegetative structure [101,103]. Nowadays, the intensive (400–600 trees/ha) and the super-intensive (1600–2000 trees/ha) methods of olive growing are substituting for traditional systems in several Italian regions, causing an increase in *P. vitrealis* infestation number and intensity [103,104]. These management systems, together with advantageous climatic conditions, allow the insect to carry on continual generations during the year [103]. Even if is still unknown how climate change is influencing *P. vitrealis* life cycle, elevated temperatures promote high population density in the Mediterranean, especially in the autumn. Furthermore, temperature is the major climatic parameter influencing the duration of the *P. vitrealis* pupal stage [101].

### 3.2. The Olive Leaf Gall Midge, Dasineura oleae

**Figure 2:**
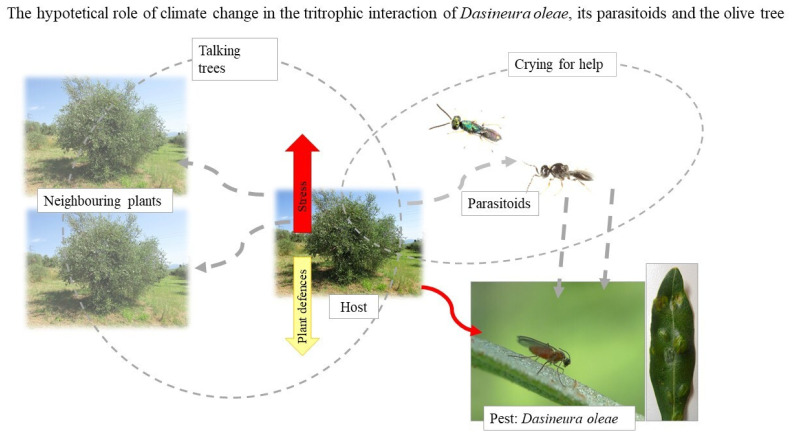
The tritrophic interaction of *Dasienura oleae* (Diptera: Cecidomyiidae), its parasitoids and the olive tree could be hypothetically altered by global heating. Under increasing temperature (atmosphere and soil), the olive tree starts suffering, and the production of secondary metabolites and other plant defensive traits (e.g., the phenomena of “talking tree” (host–host) and “crying for help” (host–parasitoids)) may be affected [2]. This may cause a disequilibrium of the tritrophic interaction and the consequent occurrence of pest outbreaks [2,100].

The recent outbreaks of *Dasineura oleae* (Angelini) (Diptera: Cecidomyiidae) in several Mediterranean regions prompted the consideration of this gall pest as a serious threat for the olive grove [100,105,106,107,108]. Little is known about this species, since before these outbreaks it has never been assumed as problematic [108]. However, leaves attacked by *D. oleae* are physiologically compromised, and premature foliar dropping is recorded when serious infestations occur, prompting yield losses to be hypothesized [106,107]. The causes of *D. oleae* outbreaks are currently unknown. However, Simoglou et al. (2012) [100] speculated about the importance of climate change on this phenomenon, since alterations of the climate parameters may affect several aspects of the pest (e.g., number of generations, abundance) and its natural enemies (e.g., success of parasitization activity) [100]. This may lead to an alteration of the tritrophic interaction concerning *D. oleae*, its parasitoids and the olive tree [2], resulting in unpredictable *D. oleae* outbreaks [2,100] (Figure 2). Picchi et al. (2021) [108] stressed the importance of pest monitoring in the outbreak areas to provide useful information concerning pest control strategies for olive growers. In this way, farmers may have an overview of pest population abundance and its natural enemies, offering the chance to study the effect of climate change on crop–pest synchrony over a long period [108]. 

## 4. Conclusions

*Olea europaea* is a perennial plant having a slow vegetative growth and a long biological cycle. The estimation of its adaptability to climate change is not simple since the availability of long-term climatic data is scarce. Consequentially, the investigation concerning the influence of global heating on olive pests is complex. The use of predictive models may help in this challenging task, even if the adoption of these tools in the Mediterranean scenario is mostly only related to the key pest *B. oleae*, while few studies have been conducted on the other major olive pests (e.g., the olive moths, *D. oleae*). Scientists should consider the limits linked to the predictive model selected in order to avoid misleading interpretation of the model’s output. Moreover, to face the global climatic change, the use of preventive techniques, such as frequent pest monitoring, together with the participation of the local extension services and the use of DSS, is fundamental. According to Damos (2015) [109], the use of these tools implies less chemical adoption and replacement by eco-friendly and safe alternatives, avoiding unwanted consequences of pesticide applications that may have an influence on climate change. Furthermore, even the issue of olive orchard scale has to be considered since the dynamic pest populations, especially in the case of good fliers, can vary according to this parameter. For instance, *B. oleae* adaptive behavior is already known at the regional scale (e.g., movement from low to high altitude in function of season, food availability, climate change) [110]. According to Hamman et al. (2021) [111], future scenarios at a major scale may include distributional changes, such as expansions into new areas, especially towards the historically cooler and upper elevations. Since the olive tree cultivation range is expected to increase in currently unfavorable and cool areas, we also expect a geographical shift of olive pests. In Italy, *B. oleae* distribution under climate warming is expected to reach the coolest areas of the peninsula, namely the Po Valley and the Apennines [51]. Nowadays, the lack of knowledge on the effects of global warming on major olive pests makes the olive groves an even more fragile and vulnerable agroecosystem. Further investigations are urgently needed on the topic in order to protect the Mediterranean olive heritage.
